# A review of progress and an advanced method for shock advice algorithms in automated external defibrillators

**DOI:** 10.1186/s12938-022-00993-w

**Published:** 2022-04-02

**Authors:** Minh Tuan Nguyen, Thu-Hang T. Nguyen, Hai-Chau Le

**Affiliations:** Posts and Telecommunications Institute of Technology, Hanoi, Vietnam

**Keywords:** Electrocardiogram, Shock advice algorithm, Automated external defibrillator, Machine learning, Deep learning

## Abstract

Shock advice algorithm plays a vital role in the detection of sudden cardiac arrests on electrocardiogram signals and hence, brings about survival improvement by delivering prompt defibrillation. The last decade has witnessed a surge of research efforts in racing for efficient shock advice algorithms, in this context. On one hand, it has been reported that the classification performance of traditional threshold-based methods has not complied with the American Heart Association recommendations. On the other hand, the rise of machine learning and deep learning-based counterparts is paving the new ways for the development of intelligent shock advice algorithms. In this paper, we firstly provide a comprehensive survey on the development of shock advice algorithms for rhythm analysis in automated external defibrillators. Shock advice algorithms are categorized into three groups based on the classification methods in which the detection performance is significantly improved by the use of machine learning and/or deep learning techniques instead of threshold-based approaches. Indeed, in threshold-based shock advice algorithms, a parameter is calculated as a threshold to distinguish shockable rhythms from non-shockable ones. In contrast, machine learning-based methods combine multiple parameters of conventional threshold-based approaches as a set of features to recognize sudden cardiac arrest. Noticeably, those features are possibly extracted from stand-alone ECGs, alternative signals using various decomposition techniques, or fully augmented ECG segments. Moreover, these signals can be also used directly as the input channels of deep learning-based shock advice algorithm designs. Then, we propose an advanced shock advice algorithm using a support vector machine classifier and a feature set extracted from a fully augmented ECG segment with its shockable and non-shockable signals. The relatively high detection performance of the proposed shock advice algorithm implies a potential application for the automated external defibrillator in the practical clinic environment. Finally, we outline several interesting yet challenging research problems for further investigation.

## Introduction

The sequence of interventions that must occur in rapid success to maximize the chain of survival from SCA, known as the ‘Chain of Survival’, has been adopted by the AHA and other health organizations in the world since the early 90 s. Such the chain of survival consists of four links including early recognition, early CPR, early defibrillation, and early basic life support [1].

As a key factor of the chain, oxygenated blood flowing to the brain and other vital organs can be kept by CPR and then SH rhythms representing an SCA can be detected by an AED. Survival in presence of SH rhythms including VF/VT is reduced by nearly 10% for each minute of defibrillation delay. However, such the ratio can he chest compression pauses. Here, the onsets/offsets of the pauses based on the envelope of the thoracic impedance signal, which is collected from the defibrillation pads, have been addressed [5]. For such method developments, non-public databases including CPR artifacts are necessarily collected across diverse sources, posing many difficulties in the performance comparison of broad scenarios. Furthermore, a huge amount of time and expense for the data collection in real environments also become great challenges for medical experts.

In mitigating the difficulties associated with the data collection, four ECG databases without CPR artifacts, which are CUDB, VFDB, MITDB, and AHA databases, are provided publicly for the development of new detection algorithms. In addition, the importance of SAA designs utilizing clear-artifact ECG databases is to upgrade the current SAAs in practical reliable AEDs, which require the CPR interruption for rhythm analysis to diagnose the SCA. Over the last 15 years, conventional methods using public databases include extraction of one or a few parameters, which are then analyzed to figure out the most common characteristics in terms of SH/NSH rhythm classification. Thereafter, an individual threshold corresponding to the extracted parameters is constructed to form a threshold-based SAA [6]. Moreover, recent developments of rhythm analysis focus on the design of intelligent SAAs employing ML and/or DL techniques. The basic principle of ML-based SAAs is to search for the most informative combination of features, which is identified by FS algorithms, FV procedures, and a ML classifier. For DL-based SAAs, CNNs can be implemented as a complete algorithm [7] or a CNNE to produce a set of deep features on a pre-selected layer, which is then fed into appropriate ML classifiers [8].

Generally, intelligent ML-based SAAs outperform threshold-based ones because a large number of Supplementary algorithms can be deployed to calculate necessary ICFs for their ML classifiers. Indeed, various threshold-based methods are compared their SCA detection performance in [7], i.e. VF filter, standard exponential algorithm, modified exponential algorithm, complexity measure algorithm, threshold crossing intervals algorithm, auto-correlation algorithm, and spectral algorithm. All of these methods use an individual threshold to determine the SCA presence on the ECG segments. Taking an example, the SH ECG segment is assumed as a quasi-sinusoidal waveform, which is combined with its copy shifted by half a period. VF filter outcome then shows low or high amplitude for the SH or the NSH segments. Consequently, a threshold equal to 0.625 is constructed to detect the SCA based on the amplitude of the VF filter outputs. Most of the algorithms are now used individually for the extraction of ICFs, which are then fed into different ML classifiers. Furthermore, intelligent DL-based SAAs also produce a better diagnosis performance in comparison with the threshold-based SAAs. It is because employing DL techniques can help to learn successfully the characteristics of input signals in terms of SH/NSH rhythms through their layers. Moreover, well-founded performance results are produced frequently by the FV applying the statistically valid manners such as tenfold CV and fivefold CV in [7, 8], which are hardly seen in conventional methods. The validation data are separated randomly into different folds for which one fold is for model testing and the others are used as training data. The CV procedure is repeated with other folds to ensure every single fold becomes the testing data one time. The purpose of the CV methods is to validate the model stability with different amounts of training and testing data. The mean and standard deviation are computed to represent the average performance of the proposed models.

Obviously, the common objective of the studies is to figure out effective designs of rhythm analysis for SAAs in order to improve certainly the SH/NSH rhythm classification performance, which needs to be over the minimum requirement of the AHA recommendations [9]. However, the SAA performance is still needed to be further improved for reducing the inappropriate diagnosis, which causes certainly the physical harms or unexpected deaths for the patients if the irrelevant defibrillation is provided. Precisely, incorrect diagnosis for the patients who are actually under SCA leads to no electrical shock provided. Otherwise, the patients, who have no SCA, are given the countershock, which causes definitely the artificial SCA and put the patients into life-threatening situations [8, 9]. It is noteworthy that these methods can be applied for SCA detection during the ventilation intervals or chest compression pauses. Moreover, linking to the reliably adaptive filters to obtain the clear-artifact ECG, which is then used as the input signals, allows these methods to diagnose the SCA during the CPR in and out of hospital environments.

It is clear that a survey of the SAA designs in terms of performance and technique is absolutely helpful as a starting point for forward-looking SAA research developments. Therefore, the state-of-the-art rhythm analysis methods using public ECG databases are reviewed thoroughly for practical AEDs. We believe that our survey can be a motivation for further contributions to shorten the path to practical AED implementations. In general, in terms of the technique utilized, SAA designs can be categorized into three types including threshold-based, ML-based, and DL-based SAAs. For the first category, the parameters are computed and compared to decide whether the rhythm is SH or NSH. A set of parameters are used as the input features for ML classifiers in the second category for the SH/NSH rhythm classification. The features are learned automatically through multiple layers during training process in the last category, which requires neither FE nor FS algorithm unlike that in the second category.

In addition, to further enhance the performance of SAAs for SCA diagnosis, we propose an intelligent SAA that utilizes a fully augmented ECG segment with its SH and NSH signals for the FE. Finally, we discuss possible future research problems for the design of intelligent SAAs.

The rest of the paper is organized as follows: The overview of rhythm analysis is described in the next Section, followed by An Advanced Method Proposal Section. New design strategies of rhythm analysis for intelligent SAA are shown in Section of Research Opportunities, while “Conclusion” Section presents the significance of our review for future research.

## Overview of rhythm analysis

Research on the SAA designs has been developed focusing on characteristic analysis of VF/VT rhythms, which can be used to discriminate SH/NSH rhythm. Hence, we provide the criteria used for the selection of typical references in this review. Firstly, the selected articles focus on the designs of SAA for the AED over the last 15 years using the public ECG databases to diagnose SH/NSH ECG segments. It is noteworthy that another research topic concentrates on the performance improvement of the SAA designs during CPR. In other words, the method development of the SAA designs in these works uses the ECG database with CPR artifacts, known as chest compression signals, which are the nonpublic ECG databases. Hence, the topic related to SAA designs during CPR will not be included in this review. Secondly, ML and DL techniques included in the development procedures of the SAA designs are employed in the selected articles. The studies adopted the threshold-based methods are also collected for this review to emphasize the evolution of the SAA designs. Finally, AHA recommendations have been used commonly as the reliable criteria for the estimation of the practical AED performance. Therefore, we choose these recommendations for classification performance comparison of the SAA designs shown in collected publications.

We classify the SAA designs using the public ECG databases into three categories based on the state-of-the-art methodologies for the comprehensiveness of review purpose as follows:

- Threshold-based SAAs;
- Intelligent ML-based SAAs;
- Intelligent DL-based SAAs.

**Fig. 1:**
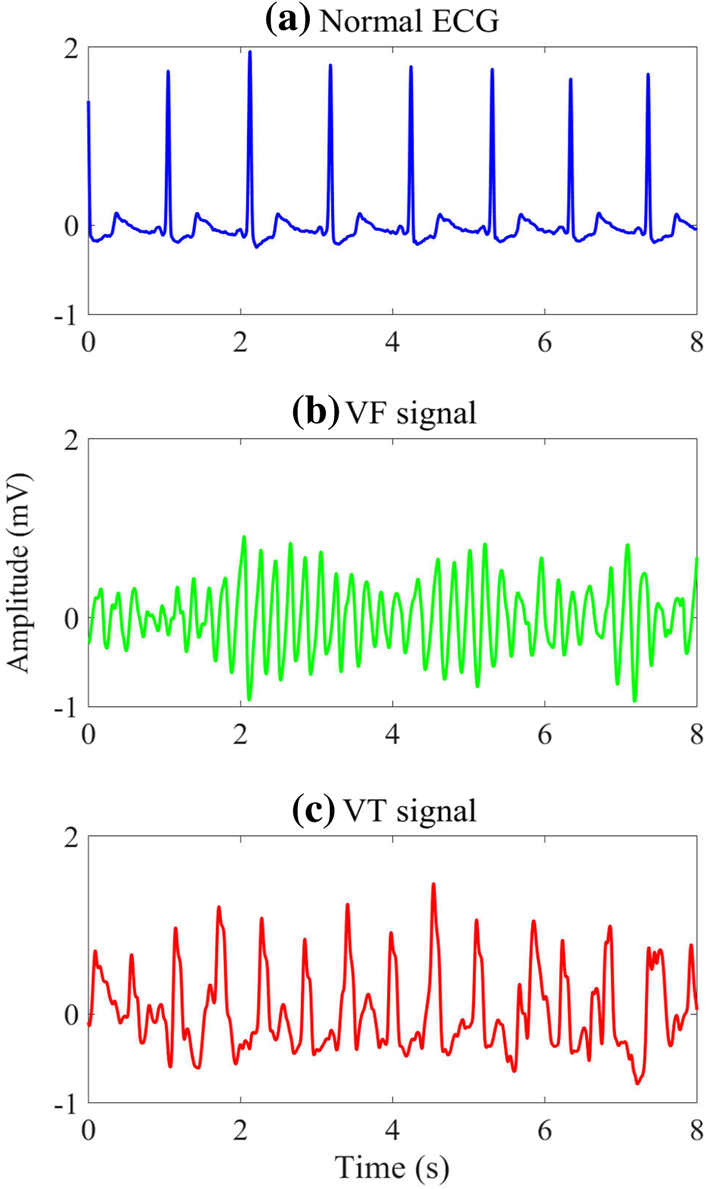
Examples of **a** normal ECG, **b** VF and **c** VT signal

Fig. 1 shows an example of the normal ECG segment, VF, and VT signals. The main purpose of the AED is to identify correctly normal ECG from VF or VT signals for further life-support decisions.

### Threshold-based SAAs

**Fig. 2:**
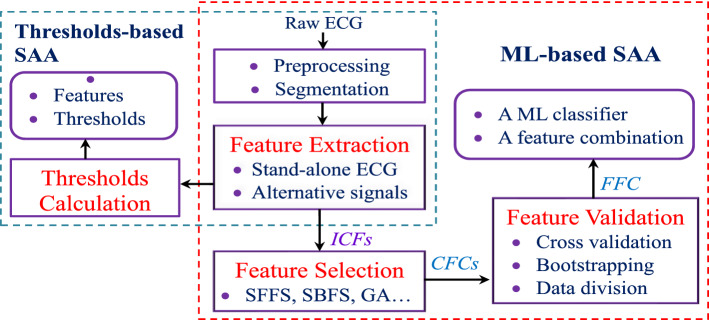
The development procedures for the threshold- and the intelligent ML-based SAAs

The development procedure using the thresholds largely depends on SH rhythm analysis. A summary of the development procedures for the threshold-based SAAs in comparison with that of the intelligent ML-based SAAs is shown in Fig. 2. Particularly, to investigate the dynamic law or random behavior of the ECG signal, the authors of [10] proposed the time delay algorithm, which counts the number of boxes on a grid filled by ECG and its time delay signals. The threshold is set as the proportion between the number of filled boxes and all boxes on the grid. Here, the VF signal irregularly fills the boxes with a large number of visited boxes while the NSH signal shows a regular behavior with only a small number of boxes filled. In [11], the threshold crossing sample count algorithm is based on the assessment of the proportion of time in which the ECG signal remains outside a certain threshold, which represents a baseline of the NSH segment or mean value of the VF signal. An alternative method in [12] suggests that the time in which the VF signal remains outside the threshold is significantly larger than that NSH signal does. Moreover, the QRS complex is significantly wider for the VT signal compared to that of the NSH signal while the VF signal has no QRS complexes. Therefore, the mean absolute value of the NSH signal is low for most of the time, while this value is comparatively large for the SH signal. This is because that SH signal hardly goes through the baseline and the absolute amplitude of the NSH signal is low for most of the time. Moreover, the degree of similarity between the VF signal and its first 2 intrinsic mode functions using EMD technique is also exploited to assign the VT with a rate lower than 180 beat per minute as the NSH rhythm. The authors of [13] measure the correlation represented by 2 angles of 2 pairs of vectors using the EMD technique. For the VF signal, the first angle between ECG signal and its first IMF is very small while the second between ECG signal and its residual is close to 90 degrees. The revert trend is shown for a non-VF signal with a large value for the first and a small value for the second angle. An effective threshold of approximate entropy is generated directly from the first intrinsic mode function of the stand-alone ECG [14] while the use of analysis model for the threshold construction improves certainly the performance in terms of VF/VT detection [15].

The most important advantage of the threshold-based SAAs is the simplicity with only one or few parameters and appropriate thresholds. The quality of parameters extracted from stand-alone ECG or decomposed signals is also crucial in terms of the reliability and performance of the proposed algorithm. It is always desired to extract the most representative, critical, and relevant parameter, which requires deep domain knowledge and expertise. However, a statistically valid manner has not been attempted for development procedure. Hence, it is hard to say that the threshold-based SAA is reliable when applying for practical AED with real-time data. Table  1 shows a summary of threshold-based SAAs for rhythm analysis. All methods report that Se is significantly lower than 90%, but Sp is well above 95%, which are the minimum values suggested by the AHA for AED performance [9]. The Sp is more important than the Se because no patient should be delivered electrical shock due to an incorrect diagnosis of AED, which may cause an artificial cardiac arrest and put patient into danger. However, low Se results in the delay of proper defibrillation, which decreases definitely the chance of survival.

**Table 1:** Summary of the threshold-based SAAs

Refs., Year	Database	Signal for FE, Segment length	Method description	Feature	Limitation	Key Findings
[10], 2007	MITDB, CUDB, AHA	Stand-alone ECG, 8 s	Counting number of boxes on a grid filled by ECG and its delay signals.	Phase space reconstruction.	No validation. Low performance.	Phase space reconstruction parameter and a threshold. VF and NSH signals show irregular and regular behaviors.
[11], 2009	MITDB, CUDB	Stand-alone ECG, 8 s	Assessment of the proportion of time in which the ECG signal is above a certain threshold.	Threshold crossing sample count.	No validation. Low performance.	Threshold crossing sample count parameter and a threshold. The time in which VF signal remains outside the threshold is larger than that in which NSH signal does.
[12], 2010	MITDB, CUDB, VFDB	Stand-alone ECG and intrinsic mode functions using EMD, 8 s	Calculation of mean absolute value of the signal for SH/NSH rhythm classification. Calculation of differences between ECG and first 2 intrinsic mode functions. Normalized mean absolute value for VF/VT classification.	Mean absolute value and normalized mean absolute value	No validation. Requirement of 2 algorithms. Low performance.	SH signal hardly goes through the baseline and low absolute amplitude of the NSH signal for most of the time. Similarity between VF signal and its first 2 intrinsic mode functions.
[13], 2011	MITDB, CUDB, VFDB	Stand-alone ECG and intrinsic mode functions using EMD, 8 s	Measurement of correlation between ECG signal and its first intrinsic mode function, residual using the angles to form a complex decision parameter.	Complex decision parameter	No validation Low performance.	Complex decision parameter and a threshold. Correlation between ECG signal and its first intrinsic mode function and residual.
[15], 2012	CUDB, NSRDB	Stand-alone ECG, 4 s	Extraction of 3 features using semantic mining algorithm. Construction of thesholds based on ANOVA model.	Natural frequency. Damping coefficient. Input signal.	No validation Low performance.	Semantic mining algorithm for feature extraction. Analysis model for threshold construction.
[14], 2012	CUDB, VFDB	Stand-alone ECG and intrinsic mode functions using EMD, 10 s	Intrinsic mode function with EMD. Approximate entropy of first intrinsic mode function.	NA	Consideration of first intrinsic mode function. No validation Low performance.	Approximate entropy threshold.

### Intelligent ML-based SAA

Rhythm analysis of the SAAs using ML techniques, as demonstrated in Fig. 2, is developed with a procedure of FE, FS, and FV phases. These methods also require a set of informative ICFs in which each ICF is an individual parameter used to design threshold-based SAA. Intuitively, all the ICFs seem to be too complex and the performance of the detection algorithm using ML can be improved by the removal of irrelevant features among ICFs. Therefore, selection algorithms are certainly employed to identify the most informative CFCs. However, there are still algorithms, which are designed without the FS because the number of used ICFs is relatively small [16–19]. The performance of the ML classifiers using the CFCs is then validated. The rationale behind the use of validation is to see how well the ML model fits the input data. A few existing methods are not implemented with a reliable validation procedure [17, 18, 20–28]. An FFC and its corresponding ML classifier, which produces the highest performance, become the proposed SAA for the application in practical AED. Different input signals used for recent ML-based SAAs are summarized in Tables 2, 3, and 4.

The SAA can be explained at first by observing the input data for the FE and classification. If the input data are pre-processed/segmented only, we call it as standalone ECG, if another input data with certain transformation or decomposition is used, then we name it as alternative signals, or if both stand-alone ECG and alternative signals are employed for the input, we address them as augmented signals. The EMD, DWT, VMD, MVMD, etc. techniques are employed to derive these alternative signals. These stand-alone ECG, alternative signals, and augmented signals are explained as follows

#### Stand-alone ECG

FE from stand-alone ECG signal has been used in [20–26, 29–37]. More precisely, the authors of [20] and [23] use the linear discriminant analysis and the NEWFM, which embedded the FS, to select the FFC of 4 and 11 features among 10 and 15 ICFs, respectively. In addition, Alonso-Atienza *et* *al*. [21] suggest an alternative method including the SVM and the SBFS in combination with bootstrapping procedure. The highest performance of SVM classifier using an FFC of 9 features among 13 ICFs on testing data shows that the FS significantly eliminates the irrelevant features. A set of temporal, spectral, and time-frequency features are extracted and then selected by the bootstrap re-sampling procedure in combination with ML classifier [26]. These methods apply the FS for the selection of the most informative feature combination but do not use any validation procedures for estimation of the statistical performance of the ML classifiers and the FFCs. Noticeably, the classification performance of the proposed SAA in [20] and [23] do not meet the AHA recommendations.

The authors of [29, 30, 32–37] use all 3 phases for the development of intelligent SAA. The FS based feature ranking approaches, which are SBFS in combination with the bootstrapping procedure [29], the SVM performance using individual ICFs [30], GA [32, 33], GRAE [34], SVM with bootstrap re-sampling [35], Gaussian GA [36], and differential evolution algorithm [37], are applied to select the most informative CFCs among all ICFs. The validation procedures are also implemented for the ML classifiers using the CFCs such as bootstrapping [29], record-based data division, and data-based data division [30], five-folds CV [32, 33, 35], 20-folds CV [34], 3-folds CV [36], and 10-folds CV [37]. These methods show relatively high performance for the SVM classifiers using FFC of 30, 3, 2, 4, 13, 3, 25, and 3 features in [29, 30, 32–36], and [37] respectively, which meets the AHA recommendations. However, the comparison of validation performance between the FFC and a combination of all ICFs is not shown because no validation for all ICFs [30, 32, 33]. Hence, the effectiveness of the FS is still questionable. In addition, the highest performance of the C4.5 classifier using all ICFs implies that the FS namely GRAE [34] is less effective than the others suggested in [29, 30, 32, 33, 35–37].

In [31], a RF classifier is used and validated by 10-folds CV using all of 17 ICFs for different segment lengths. The best performance is reported for overlapping 8 s segment length and slightly above the recommendation of AHA. However, the performance of feature subsets is not estimated due to the absence of the FS. The performance of the SVM classifier using the combination of 11 features shows better classification than that of the threshold-based method namely the VF-filter algorithm for both VF/non-VF and SH/NSH rhythm scenarios [22]. The time-delay algorithm is used to extract a feature set, which is effective to classify different rhythms such as VF/non-VF, atrial fibrillation/non-atrial fibrillation, premature ventricular contraction/non-premature ventricular contraction, and sinus rhythms [24]. QRS complex of 200 data points is computed for extraction of 7 features as the input of different ML classifiers for which the DECORATE model shows the highest performance [25]. However, no FS and FV provided in [22, 24, 25] makes it difficult to estimate the performance in a statistical manner. Table 2 shows a summary of intelligent ML-based SAA using stand-alone ECG as the input signal.

**Table 2:** Summary of intelligent ML-based SAA using stand-alone ECG

Refs., Year	Database	Signal for FE, Segment length	Method description	ICF	FS	FV	Limitation	Key Findings
[20], 2007	CUDB, VFDB, AHA	Stand-alone ECG, 10 s	Use of 10 ICFs as the input of Linear discriminant analysis to select an FFC of 4 features.	10	Linear discriminant analysis embedding FS	NA	Limited number of ICFs.	FFC of 4 features.
							No validation.	Prediction of success of defibrillation.
[25], 2011	MITDB	Stand-alone ECG, QRS comlex of 200 points	Performance comparison of KNN, neural networks and ensemble based methods.	7	NA	NA	Limited database.	Better performance of DECORATE model than others.
							Limited number of ICFs.	
							No FS and FV	
[21], 2014	CUDB, VFDB, MITDB	Stand-alone ECG, 8 s	SBFS including SVM and boots trapping to select an FFC of 7 features on training data.	13	SBFS using SVM and boostrapping	NA	Limited number of ICFs.	FFC of 9 features.
			Performance of SVM using a FFC on testing data.				No validation.	
[22], 2012	CUDB, VFDB, MITDB		Extraction of 11 Vleak features.	11	NA	NA	Limited number of ICFs.	FFC of 11 features and SVM.
			Comparison performance of SVM and VLeak threshold for VF/non-VF and shock/non-shock.				No FS and FV.	Better performance of SVM than Vleak.
[23], 2014	CUDB		Use of Hilbert transforms for peak extraction, phase space reconstruction, time domain analysis.	15	NEWFM embedding FS.	NA	Limited database.	FFC of 11 features.
			NEWFM embedding FS to select an FFC of 11 features.				No separated data for FS and testing.	
							No validation.	
[29], 2016	CUDB, VFDB, AHA, OHCA		SBFS including 2 ML classifiers and bootstrapping to select 2 CFCs.	30	SBFS using Logistic regression, Boosting and boostrapping.	Bootst-rapping	Lower validation performance of CFCs than all ICFs.	Large number of ICFs.
			Validation of CFCs and a combination of all ICFs using 5 ML classifiers and bootstrapping.					OHCA data requires two times more features than public data.
[30], 2017	CUDB, VFDB, MITDB		SVM to rank 26 ICFs and selection of 19 features.	26	Feature ranking with SVM	Record-based data division.	No validation for all ICFs.	FFC of 3 features
			Validation of every combination of 19 features using SVM and random data division.			Database-based data division.		
[24], 2018	CUDB, VFDB, MITDB, AFDB		Algorithm design for classification of VF/non-VF, Atrial fibrillation/non	6	NA	NA	Limited number of ICFs.	Effective features computed from time-delay algorithm.
			-Atrial fibrillation, premature ventricular contraction/non-premature ventricular contraction, and sinus arrhythmia.				No FS and FV.	
			SVM and Bayer decision tree for VF/non-VF classification.					
[31], 2016	CUDB, VFDB, MITDB		Use of RF and 10folds CV to validate the combination of all ICFs for different window lengths.	17	NA	10-folds CV	No FS	Best performance for overlapping 8 s-segment.
[32], 2014	CUDB, VFDB, MITDB	Stand-alone ECG, 5 s	GA based feature ranking.	14	GA	five-folds CV	-Limited number of ICFs.	FFC of 2 features.
			Performance investigation of every combination of 9 features using SVM.				No validation for all ICFs.	
			Validation of 9 combinations using five-folds CV and SVM.					
[33], 2018	CUDB, VFDB		GA based feature ranking for selection of 7 good features.	11	GA	Five-folds CV	Limited number of ICFs.	FFC of 4 features.
			Performance estimation of SVM using every feature combination of good features.				Only 1 classifier	
			Validation performance of SVM using 6 combinations with five-folds CV.				Similar method of [15]	
[34], 2018	CUDB, VFDB		C4.5 for classification of normal, VF, and VT segments	13	GRAE	20-folds CV	Highest performance of all ICFs	Identification of confidence factor value of C4.5
			Feature ranking using gain ratio attribute evaluation.					
			Investigation of different confidence factor for C4.5					
[37], 2021	MITDB, CUDB,VFDB		Application of SVM and AdaBoost for the FS based differential evolution algorithm and classification of VF and non-VF rhythms.	17	Differential evolution algorithm	10-folds CV	Only SVM.	Effective AdaBoost for data weight assignment to improve SVM classification performance.
			Extraction of 17 conventional features and selection of 3 as the optimal feature subset				Limited number of ICFs.	
							No validation for all ICFs.	
[35], 2012	VFDB, AHA	Stand-alone ECG, 1.024 s	Extraction of temporal, spectral, and time-frequency features.	37	SVM-bootstrap resampling SVM-recursive feature elimination Filter methods	5-folds CV	Performance analysis based on 1 ML classifier.	Highest performance of FFC of 3 features.
			Comparison of SVM-bootstrap resampling, SVM-recursive feature elimination and filter methods.				Only 1 s-segment	Efficient SVM-bootstrap resampling.
[26], 2012	VFDB,AHA		Extraction of temporal, spectral, and time-frequency features.	27	Bootstrap resampling	NA	Performance analysis based on a ML classifier.	Self organizing map using an FFC of 11 features.
			Selection of 11 features using bootstrap resampling based feature selection.					
			Self organising map for classification.					
[36], 2019	CUDB, VFDB, NSRDB	Stand-alone ECG, 3 s	Investigation of 47 time domain and wavelet features. Selection of 25 by FS.	47	Gaussian GA	3-folds CV	Investigation of a classifier.	17 wavelet features (out of 25) show the significant efficiency of wavelet method.
			Detection of VF/VT and normal ECG segment by the first SVM classifier.				Validation on only a database.	Practical application for AED processor.
			Discrimination between VF and VT by the second SVM classifier.				Low average CV performance	Reletive short of segment length.
			Hardware implementation of the proposed algorithm for the AED.					

#### Alternative signals

An effective strategy to improve the classification performance of the proposed SAAs for the AED is the increase in the quality of the extracted ICFs using various decomposition techniques. Indeed, the sub-signals allow investigating new ICFs, which contribute definitely to the improvement of the final detection performance. The summary of the ML-based SAA using alternative signals is given in Table 3.

**Table 3:** Summary of intelligent ML-based SAA using alternative signals

Refs., Year	Database	Signal for FE, Segment length	Method description	ICF	FS	FV	Limitation	Key Findings
[16], 2016	CUDB, MITDB, AHA	Subsignals using DWT, 3 s	Use of DWT for reconstruction of subsignals.	1	NA	Five-folds CV	Insignificant improvement for classification performance.	Only a feature and SVM classifier.
			Calculation of the number of samples which is larger or smaller than positive or negative thresholds during 1 s segment.				Time consumed for FE may be over segment length of 3 s.	Relative short of segment length.
			Use of average numbers of samples as a feature for SVM classifier.					
[27], 2017	MITDB	Subsignals using DWT, 5.7 s	5 levels of wavelet coefficients using DWT.	20	NA	NA	Limited database.	Peak extraction from wavelet coefficients.
			Peak extraction from wavelet coefficients, plotted in 3D PRS.				No FS and validation.	
			NEWFM classifier using 20 features considered as distances between origin of coordinates axis and peaks.					
[40], 2017	CUDB, VFDB, MITDB	Subsignals using DWT, 10 s	DWT with 4 levels-decomposition.	31	SFFS	Five-folds CV	Only 1 classifier.	FFC of 10 features.
			Feature extracted from wavelet coefficients.				No validation performance for all ICFs.	The best ranking method of ReliefF.
			SFFS to select 14 features.					
			Feature ranking using 6 methods for set of 14 features.					
			KNN classifier using different sets of features of 6 ranking methods.					
[38], 2019	CUDB VFDB, MITDB	Subsignals using DWT, 5 s	Performance comparision of C4.5 and SVM for detection of VF, VT.	24	GRAE	10-folds CV	Highest performance of all ICF.	Generation of signals concentration on VT and VF components based on DWT.
			Using DWT as low-pass and high-pass filters for generation of alternative signals.				Ineffective FS.	
			Features extracted from alternative signals.					
[39], 2016	CUDB, VFDB, MITDB	Subsignal using wavelet decomposition, 2 s	Analysis on wavelet decomposition to design an optimal low-pass filter showing a minimum stopband ripple energy.	12	NA	10-folds CV	Limited number of ICFs.	Selection of six subsignals based on orthogonal conditions.
							No FS.	Productive SVM for SH rhythm detection.
								Relative short of segment length
[41], 2016	CUDB, VFDB, MITDB	Modes using VMD, 5 s	5 modes using VMD.	9	FS based feature scoring	Five-folds CV	Limited number of ICFs.	Modes using VMD for FE.
			FE from first 3 modes.				Hand-picked data.	FFC of 7 features.
			The FS based feature scoring to select an FFC of 7 features.				Random reconstruction of modes.	
			Validation of the FFC using RF and five-folds CV.					
[43], 2017	AFDB MITDB, NSRDB	Modes using VMD, 8 s	Decomposition of ECG into 5 modes.	20	NA	Five-folds CV	No FS.	Effective entropy features.
			Sample entropy and distribution entropy of modes.				Hand-picked data.	High performance of SVM with KBF kernel among others.
			Performance of 2 ML classifiers for normal, AF, and VF scenario.				Random generation of modes.	
							Limited number of ICFs	
[42], 2018	CUDB, VFDB, MITDB	Modes using adaptive VMD, 5 s	5 modes using adaptive VMD.	30	NA	10-folds CV	No FS.	Optimal parameters for adaptive VMD.
			10-folds CV for Boosted CART using all ICFs.				Simple selection of VMD parameters.	
[28], 2018	CUDB, VFDB,	Modes using dimensional Taylor Fourier transform, 8 s	Decomposition of ECG segment into oscillatory modes using dimensional Taylor Fourier transform.	20	NA	NA	Low performance.	New diagnostic features of magnitude and phase differences using dimensional Taylor Fourier transform.
			20-dimension feature vector based on magnitude and phase differences.				No FS and FV.	
			LSSVM classifier for detection of shock/non-shock, VT/VF, and VF/non-VF.				Only 1 classifier.	
[17], 2009	MITDB	Intrinsic mode functions using EMD, 7 s	Use of intrinsic mode function with EMD.	2	NA	NA	No validation.	Orthogonality of IMFs as the features.
			Calculation of 2 angles between first 3 IMFs for Bayer decision theory.				Limited database	
[18], 2017	VFDB AHA	Image of time-frequency, 150 ms	Construction of time-frequency image.	1	NA	NA	Only 1 feature.	Algorithm design for multiple classification using different binary ML classifiers.
			Performance comparison of different ML classifiers for classification of normal, VF, VT, and other rhythms.				No validation.	
							Complexity due to 3 ML classifiers for multiple classification	
[19], 2018	VFDB AHA	Time-frequency representation image, 1.2 s	Extraction of image using Hilbert transform and Time-frequency representation techniques.	1	NA	Five-folds CV	Only 1 feature.	Effective feature of TFRI image.
			Use of multiple ML classifiers to detect normal, VF, VT, other rhythms.				Increase in complexity due to binary algorithms for multiple classification	Hierarchical topology of 3 ML classifiers.

The DWT is applied to decompose the ECG segment into different sub-signals with wavelet coefficients [16, 27, 38–40], which are corresponded to the number of bandwidths emphasizing both SH and NSH rhythms. According to a comparison shown in [16], the validation performance is not significantly improved in comparison with that of [21]. However, the complexity of the FE is reduced because only one feature namely the average number of samples computed from every 1 s-segment of the input ECG signal is used as the input of the SVM classifier [16]. All of 20 ICFs, which are the distances between the origin of coordinates axis and peaks extracted from wavelet coefficients using a three-dimensional (3D) phase-space reconstruction diagram, are suggested as the input of the NEWFM classifier without the FS and the FV during the development procedure [27]. An important use of the DWT is given in [38], which considered DWT as the low-pass and high-pass filters to generate coefficients emphasizing VF and VT components. As a result, alternative signals are produced for extraction of a feature set used as the input of different ML classifiers. Another design of the low-pass filter, which minimizes the ripple energy in the stop-band, is proposed in [39] using the wavelet decomposition technique. Then, a filtered signal including the SH components is generated by such filter to extract features for further classification by ML algorithms. The main purpose of [40] is to investigate the performance of various ranking methods related to the selection of informative feature set. Indeed, the highest validation performance is produced for the KNN classifier using a FFC of 10 features, which is ranked by the ReliefF method.

The authors of [41–43] use 5 modes decomposed by the state-of-the-art technique, namely VMD, for the FE. The observation about waveform and frequency of modes results in the utility of the first 3 modes for extraction of 9 features such as energy, renyi entropy, and permutation entropy. Indeed, the P-wave, the QRS-complex, and the T-wave of ECG included grossly in first 3 modes are the main diagnostic components for the SH/NSH classification [41]. Moreover, analysis of the data fidelity constraints, which show the decrease in energy of the original ECG signal in the high frequencies of decomposed signals, is implemented in [42]. Similarly, ventricular arrhythmia components are captured in the first 5 modes using VMD techniques [43]. Obviously, the modes with low frequencies, which contain much information of the original ECG segments, are suggested as the efficient sub-signals for the FE in these studies. This means a large number of modes can be used for the FE rather than only first 5 modes in original ECG decomposition. A set of 7 relevant features as the FFC are selected by the FS based feature scoring algorithm in [41], while no FS is given in [42] and [43] considering the ICFs of 20 and 30 features, respectively. The ML algorithms such as RF, Boosted-CART, and SVM are used as the classification methods in [41–43], which also employ hyper-parameter optimization methods to search the optimal ML models for the SCA designs. The detection performance is slightly higher for the proposed algorithm in [42] than that in [41]. This is caused probably by the irrelevant features, which should have been eliminated by an effective FS algorithm. A 20-dimensional Taylor Fourier transform feature vector of magnitudes and phase differences of the modes are used as the input of the least square SVM in [28]. Here, the authors focus on analysis and comparison of the least square SVM using different kernels such as linear and radial basis functions. The diagnosis performance of the least square SVM does not meet the AHA recommendations, which suggest an ineffective feature set constructed by the dimensional Taylor Fourier transform.

Another method, which exploits the orthogonality by using 2 angles of the first 3 IMFs as the input features of Bayer decision theory using the EMD technique, is proposed in [17]. The performance of the proposed algorithms is close to 100% for both Se and Sp, which may be caused by the use of small input data. Moreover, no statistically valid manner is applied for the development of the proposed algorithms. In [18], an image is created by applying the Time-frequency representation for the input ECG signal. The hierarchical topology of different ML classifiers using such image is proposed for multiple classifications of normal, VF, VT, and other rhythms. Moreover, the image is upgraded with both Hilbert transform and Time-frequency representation for the use as the input of hierarchical topology of the ML classifiers in terms of classification performance [19]. However, the complexity of such proposed algorithms are increased due to the use of binary algorithms for multiple classification and signal processing techniques for image generation.

#### Augmented signals

For the stand-alone ECG segment or transformed, decomposed signals, which are considered for the FE, it is noteworthy that each signal may carry a similar amount of information regarding SH rhythms. We may try "augmentation" of stand-alone ECG with the decomposed signals for the FE. The detection performance of SAA for SH/NSH rhythms is improved when the augmented stand-alone ECG segment with its NSH signal is used for the investigation of a new ICF set. Indeed, the authors of [44] modify the VMD technique to generate various modes from the original ECG segment with pre-selected center frequencies. Then, the sum of the modes with center frequencies over 10 Hz is considered as the NSH signal, which includes most of normal components from the original ECG segment. The SAA is proposed with an FFC of 20 features, extracted from both stand-alone ECG and its NSH signal, and SVM classifier. However, the FS is time-consumed due to the dual-layers of the GA and SFFS for the selection of the FFC. The Stationary wavelet transform is applied for the stand-alone ECG segment and its square to generate a total of sub-signals on which 24 ICFs of sample entropy are computed [45]. The FFC of 10 features is addressed with the KNN classifier for the VFVT/non-VFVT scenario using NTscore for the bandwidth ranking. Clearly, no consideration of conventional features other than sample entropy may cause a limitation of the classification performance. Table 4 shows the summary of existing SAAs using the augmented signals as the input of the ML classifiers.

**Table 4:** Summary of intelligent ML-based SAA using augmented signals

Refs., Year	Database	Signal for FE, Segment length	Method description	ICF	FS	FV	Limitation	Key Findings
[44], 2017	CUDB, VFDB	ECG segment, NSH signal using MVMD, 8 s	Reconstruction of NSH signal using MVMD.	54	GA and SFFS using 3 ML classifiers and fivefolds CV.	Fivefolds CV	Limited database.	FFC of 20 features with SVM.
			Use of both stand-alone ECG and NSH signals for FE.				Time-comsuming for FS.	Effective twolayered FS.
			The two-layered FS with 3 ML classifiers to select 3 CFCs.					NSH signal using MVMD.
			Validation of CFCs and a combination of all ICFs using 5-folds CV.					Expansion of new ICFs.
[45], 2018	CUDB	ECG segment, (ECG segment)$^2$ using Stationary wavelet transform, 4.1 s	Calculation of sample entropy for 10 selected bands using Stationary wavelet transform.	24	Bandwidth ranking with score	NA	Limited database.	Short segment.
			Bands ranked by NT-score which is a combination of relief, gain ratio, and fisher score.				No validation.	Expansion of ICFs from ECG segment and square of ECG segment.
			Performance of 3 ML classifiers for VFVT/non-VFVT and VF/non-VF scenarios.				Limited number of ICFs	

The use of augmented signals for the FE in combination with an effective FS allows to investigate an expansion of the ICFs and select successfully the most relevant FFC. Indeed, the validated performance of the proposed ML-based SAA in [44, 45] is highest and meets the AHA recommendations for the AED among other methods shown in Tables 2, 3, and 4.

### Intelligent DL-based SAA

The first application of the CNN for the classification of SH/NSH rhythm is suggested in [7]. Here, the advantages of the proposed algorithm using a fully connected eleven-layer CNN model is that the expert knowledge-based FE, feature ranking, or score based FS algorithms, and statistical analysis are not required. However, the performance results with the Se of 95.3% and the Sp of 91.0% do not meet the AHA recommendations.

For the purpose of final detection improvement, different uses of the DL are applied, that is, the CNN learns the characteristics of shockable rhythms and then extract them from a specific layer as the feature set for the input of the ML classifier [8]. Moreover, the quality of the deep features is improved because of the multiple input channels for the primary training of the CNNE using the MVMD technique. The secondary training of the ML classifier using such features also increases significantly the final performance of the proposed algorithm. The decomposition technique is also employed in [46], namely FFREWT. Here, an ECG segment is decomposed into modes used as the multiple channels of a CNN model for the SCA detection. Clearly, the VF, VT, and normal components are included in the individual modes, which contributes significantly to the improvement of the final classification performance. The authors of [47] apply extended construction of different DL techniques for which the features are learned deeper in comparison with the previous method due to multiple DL methods. Therefore, the final detection performance of the proposed algorithm is significantly improved with a short length of segment such as 4 s. This results in better survival due to short interruption of the CPR for ECG collection in terms of SCA diagnosis. In addition, A ResNet CNN model is suggested for the SAA in [48], which investigates various ECG segment lengths. The highest detection performance is generated by the CNN model using 4 s-patient’s ECG segment. The most contribution of the work is the construction of ResNet CNN structure, which is effective for the SAA design in the AED. Indeed, model structure finding plays an important role in performance improvement. Hence, a random search-based method for hyper-parameter optimization is proposed in [49]. A set of variables such as the number of sequential CNN blocks, number of filters and kernel sizes are investigated randomly to select and rank the optimal CNN modes using the median values. The high detection performance of the best CNN model, which is selected from the ranked optimal CNN models, shows the effectiveness of the proposed hyper-parameter optimization algorithm.

A pre-selected DNN structure is suggested in [50] for the detection of VF/VT rhythms using a feature set as the input. Here, the authors apply a combination of different decomposition techniques such as DWT, EMD, and VMD to improve the quality of the ECG signals for further segmentation and feature extraction. In [51], the input ECG databases are divided into various segment lengths ranging from 3 to 10 s, which are then converted into time-frequency maps using CWT. An optimal CNN structure is selected among eight candidates using such time-frequency images as the input, which produces relatively high classification performance and meets the AHA recommendations. Table 5 shows a summary of recent SAAs using DL techniques.

**Table 5:** Summary of intelligent DL-based SAA

Refs., Year	Database	Signal for FE, Segment length	Method description	ICF	FS	FV	Limitation	Key Findings
[7], 2018	CUDB, VFDB, MITDB	Stand-alone ECG, 2 s	The 11-layers CNN for classification of SH/NSH 2 s-ECG segment.	NA	NA	10-folds CV	Only one CNN structure.	Simple full CNN.
			Validation of full CNN with 10-fold CV.				Time-consuming for validation of the full CNN.	Less complexity due to no FS and FV.
								Relative short of segment length.
[8], 2018	CUDB, VFDB	ECG segment, NSH signal, SH signal, using MVMD, 8 s	NSH, SH signals generated by MVMD.	NA	NA	5-folds CV	Time-consuming for selection of CNNE.	Improvement of LF quality due to multiple channels.
			Use of ECG segment, NSH and SH signals as input channels of CNN					Improvement of final performance due to secondary training of ML classifier.
			Grid search with nested 5-fold CV to select best structure and parameters of CNNE using ML classifiers.					No need of FE and FS.
			Validation of feature vector extracted by CNNE with different ML classifiers.					
[46], 2020	CUDB, VFDB	Modes using FFREWT, 8 s	FFREWT for ECG segment decomposition into 6 modes.	NA	NA	10-folds CV	pre-selected CNN, structure.	High performance. Effective FFREWT for generation of different modes containing VF, VT, normal components of ECG.
			First 5 modes used as the input of CNN for detection of SH and NSH ECG segment, VF and VT rhythms					
[47], 2019	CUDB, VFDB, AHADB	Stand-alone ECG, 4 s	1D parallel CNN, LSTM and ANN for classification of 4 s-ECG segment.	NA	NA	NA	pre-selected CNN, LSTM, ANN structure.	High performance.
							No validation.	Multiple DLs for deep feature extraction.
								Relative short of segment length.
[48], 2020	EMS	Stand-alone patient’s ECG, 4 s	Fully CNN architecture and ResNet CNN model	NA	NA	10-folds CV	pre-selected CNN structure.	High performance for 4 s-ECG segment.
			Classification of SH/NSH for different ECG segment length.					Relative short of segment length.
			Validation with 10-fold CV.					
[49], 2020	CUDB, VFDB, MITDB, OHCA1,OHCA2	Stand-alone ECG, 5 s	Random search based method for hyper-parameters of optimal deep CNN models using number of sequential CNN blocks, number of filters, kernel sizes.	NA	NA	NA	No validation.	Productive method for selection of a deep CNN structure with optimal hyper-parameters.
			Median values to rank the optimal deep CNN models trained with various learning rates and ECG segment lengths to select the best deep CNN model.				Time-consuming for hyperparameter optimization	Model ability related to SH/NSH classification is depended largely on hyper-parameters.
[50], 2021	CUDB, VFDB	Stand-alone ECG, 5 s	DNN using a feature set extracted from ECG segments pre-processed by DWT, EMD, VMD.	24	NA	NA	No validation.	Relative short of segment length
			Comparison to various ML classifiers				pre-selected DNN	Effective decomposition techniques for data processing.
[51], 2021	CUDB, VFDB, MITDB, AHADB	Time-frequency maps, 3 s,5 s,8 s,10 s	Conversion of ECG segments into time-frequency maps using CWT.	NA	NA	10-folds CV	Time-consuming for selection of an optimal 2D CNN structure	Relative short of segment length
			Investigation of eight 2D CNN structures.					Productive transformation method using CWT.
			Consideration of different ECG segment lengths.					

### Analysis of representative works

Generally, the classification performance of the proposed ML-and DL-based SAAs is better than that of the threshold-based SAA and meets the AHA recommendations. Moreover, the thresholds are used as the extracted features in ML- and DL-based methods. It is because the combination of different thresholds improves definitely the final detection performance of the ML- or DL-based SAA designs. Therefore, we select three representative works, which are [8, 10, 44] from categories of threshold-based SAA, intelligent ML- and DL-based SAAs, for analysis and comparison.

The threshold crossing sample count is a conventional algorithm, which assesses the proportion time for that the ECG segment remains outside a threshold. The threshold represents the isoelectric line of the NSH ECG segment or mean value of VF samples. If the value of the TCSC is less than 0.2, the signal is close to the isoelectric line most of the time, which identifies a NSH ECG segment. Conversely, the higher value of TCSC than 0.2 identifies the SH ECG segment, which is far from the mean value of samples [10].

The ML techniques are employed for the SAA designs as shown in [44]. The input signals used for the FE are the augmented ECG segments, which are the original ECG segment and NSH signal. In this work, the MVMD is adopted to generate the NSH signal from the original ECG segment. A final set of 20 features are selected by the two-layer FS from 54 input features, which are extracted from both the original ECG segment and NSH signal. The CV procedure is then applied for various ML models to estimate their performance using the above final feature set. It is obvious that the quality of input features extracted from both the original ECG segment and NSH signal. Moreover, the two-layered FS including GA and SFFS increases definitely in opportunity for selection of the best features. Better quality of the input features and the two-layer FS are definitely the key elements, which have a significant impact on the classification performance improvement.

The authors of [8] proposed the DL-based SAA designs using CNN with different input signals namely fully augmented ECG segment with its NSH and SH signals generated by the MVMD. Clearly, the utility of the DL requires no FE, FS, and feature engineering, which are the mandatory requirements for the SAA design with the ML methods. Here, the CNN is considered as the feature extractor, which produces a deep set of 100 features at the fully connected layer. Also, different ML algorithms are validated their detection performance using the CV procedure and this deep feature set. The classification performance in terms of SH/NSH rhythm of the proposed SAA in [8] is dependent firstly on the input channels, known as the original ECG segment, NSH and SH signals. The sum of various modes decomposed by the MVMD including most of SH components from the original ECG segment is the SH signal. Similarly, the NSH signal is a sum of modes containing most of the NSH components from the original ECG segment. Additionally, the best CNN structure identified as the feature extractor using a grid search and the use of deep learning features for the secondary training of the ML classifiers are the second and third reasons for the dramatic increase in the detection performance of the proposed SAA.

The CV is also used in these studies to generate reliable results of the detection performance, which make the SAA designs potential for clinical environment application. Indeed, the CV procedure is implemented for three ML models known as KNN, SVM, and Boosting to evaluate their detection performance using the optimal features [44] and deep feature set [8]. A comparison between three representative candidates of different categories is shown in Table  7. The performance of the ML- and DL-based SAA [8, 44] is higher than that of threshold-based SAA [10]. Moreover, the better accuracy of 99% is produced by the SVM classifier using the optimal feature set extracted from the original ECG segment and its NSH signal in comparison to that of 98.8% generated by the SVM classifier using the deep feature set. In contrast, the KNN and Boosting classifiers fit certainly in with the deep feature set compared to the optimal features extracted from the original ECG segment and NSH signal. Indeed, the average accuracy values are 99.1% and 99.3% of the KNN and Boosting classifiers using the deep feature set. Obviously, these values are higher than the average accuracy of 98.9% and 98.4% produced by the above classifiers using the optimal feature set.

## An advanced method proposal: fully augmented ECG

To improve the detection performance of the SH/NSH rhythms, recent studies use the signals, which are generated from original ECG, for the feature extraction [8, 44]. The MVMD has been proven its effectiveness to generate new signals, which are then used with the original ECG signal for the FE [44]. However, the SH signal, which contains most of the SH components of the original ECG segments has not been considered proper for the FE. Moreover, due to no requirement of conventional expertise-based FE and FS for the DL methods, the authors of [8] considered fully augmented ECG with its NSH and SH signals, which are also produced by the MVMD, as the input channels of the CNN model. Here, the original ECG, NSH, and SH signals have not been used for the FE, also. The deep learning methods require no conventional expertise-based feature extraction and feature selection algorithms. However, the application of deep learning for the SAA design may result in a lot of work. To cope with this, the development of an optimal algorithm to select the optimal hyper-parameters of the deep learning models is critical. Furthermore, the number of layers, hidden nodes, learning rate, momentum,.. also need to be selected carefully with different optimization methods. On the other hand, another important characteristic is that a large amount of data recommended for the training process of the deep learning method is posing a big obstacle. Motivated by the above analysis, we recognize that the detection performance of the SAA can be still increased by using the fully augmented ECG with its NSH and SH signals for the FE using different conventional algorithms. Obviously, the detection performance of the ML-based SAA and DL-based SAA is better than that of the threshold-based SAA and meets the AHA requirements while the use of the fully augmented ECG with its NSH and SH signals for the FE results in an expansive feature set, which can contribute significantly to the performance enhancement of SAAs for the AED. Hence, we have proposed a novel method using ML techniques with a feature set extracted from the original ECG, NSH and SH signals to archive better performance in terms of SH/NSH rhythm classification. Here, the databases and pre-processing approaches are utilized as those presented in [8]. The contributions of this section are summarized as follows:

- We prove that the intelligent SAA design using state-of-the-art ML technique can improve the SCA diagnosis performance compared to that shown in the previous Section of this review.
- The FE based on fully augmented ECG with both its NSH and SH signals using the MVMD technique is more effective to investigate an expansive feature set than that based on stand-alone ECG, alternative, or augmented ECG with the only NSH signal.
- The proposed SAA design for the AED using the SVM model is less complex related to the training process and better detection performance in comparison with that including DL-based algorithms.

### Method

The proposed method is composed of 3 phases as shown on the right of Fig. 2. Moreover, our method adopts a set of 6 ML classifiers for both the FS and FV namely SVM, RF, KNN, Boosting, Logistic regression, Bagging.

#### Fully augmented ECG

According to [52], the spectrum amplitude of VF/VT rhythms vanishes rapidly above 10 Hz. Moreover, the bandwidth of NSH rhythms ranging from 13 Hz to 17 Hz is used effectively in [44]. Therefore, the power of the SH and NSH rhythms are reconstructed successfully on the BWs lower and above 10 Hz as shown in [8]. In this work, SH and NSH signals are used with pre-processed ECG segments for the conventional feature extraction.

**Fig. 3:**
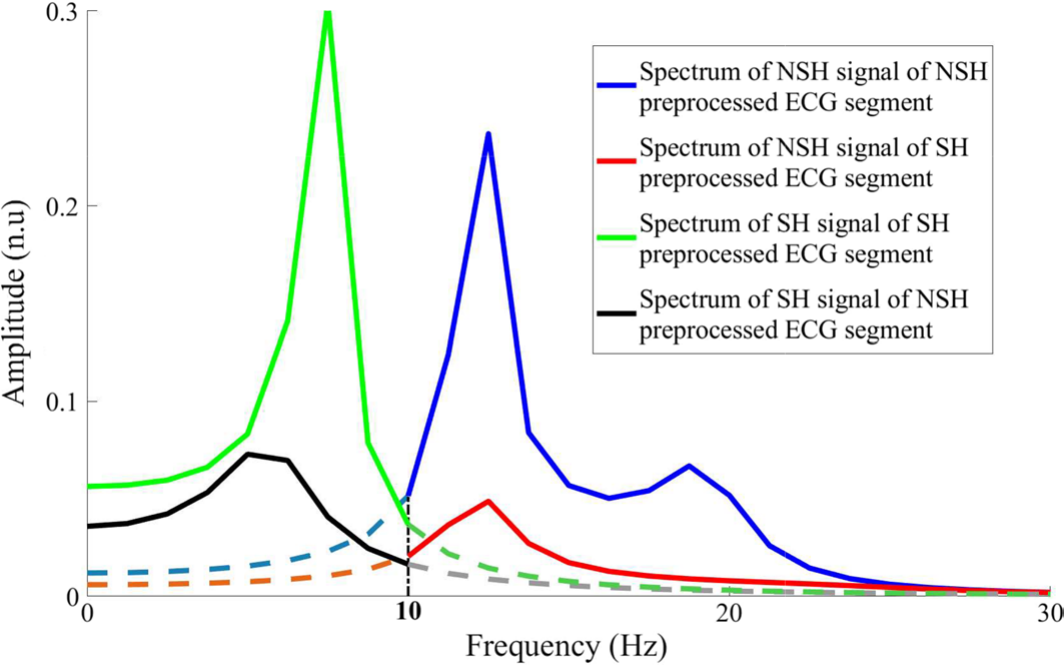
Spectrum of SH and NSH signals of the SH and NSH ECG segments

It has been proven that the total difference between the spectrums of the NSH signals for the SH and NSH ECG segments results in the reliability of this signal for feature extraction in terms of binary SH/NSH rhythm classification [44]. As can be seen from Figure  3, the power spectrum of the NSH signals is extremely different for the SH and NSH ECG segments (red and green lines) on the BW above 10 Hz. Therefore, the features, which are extracted from the NSH signal, are reliable to classify the SH/NSH rhythm. Similarly, the SH signals have significantly different power spectrums for the SH and NSH ECG segments (blue and black lines) on the BW below 10 Hz. Hence, the classification of the SH/NSH rhythm based on the features, which are extracted from the SH signal, is certainly effective.

#### Development procedure

We first use 27 ICFs suggested in [44] and 4 ICFs, which are Energy, Renyi entropy [41], Fuzzy entropy [53], and Wavelet entropy [54]. As a result, a total of 93 ICFs are extracted from the fully augmented ECG segment with its SH, and NSH signals. Then, the FS including the SFFS algorithm and the ML classifiers using Fisher score is employed to select the most relevant CFCs [55]. A CAF and CFCs are then evaluated by different ML classifiers using a 5-folds CV procedure on the evaluation data. The mean and standard deviation of the performances of the ML classifiers are computed for 50 repetitions of the 5-folds CV procedure. One of the CFCs or the CAF is selected as the FFC if its corresponding classifier produces the highest accuracy.

### Proposed SAA for AEDs

The classification performance of the ML classifiers is estimated by 4 measures, which are Ac, Se, Sp, and BER [44].

There are 6 CFCs including 38, 36, 70, 83, 91, 23 features, which are selected by the SFFS corresponding to SVM, KNN, Logistic regression, Bagging, RF, Boosting classifiers using the Fisher score. Table 6 presents the highest classification performance of the ML classifiers corresponding to each of CFCs or CAF. Obviously, the highest average accuracy is produced by the SVM classifier using the CFC of 36 features. Therefore, the SVM classifier and the CFC2 including 8 (SH_SE, SH_C2, SH_C1, SH_C3, SH_bCP, SH_bWT, SH_TCSC, SH_MAV), 12 (NSH_bWT, NSH_C2, NSH_TCSC, NSH_SE, NSH_bCP, NSH_MAV, NSH_C3, NSH_MEA, NSH_TCI, NSH_CM, NSH_C1, NSH_Li), and 16 (Lk, bCP, Hilb, C2, TCSC, PSR, SE, bWT, MAV, A2, C3, bW, MEA, C1, Li, Kurt) features extracted from the SH, NSH signals, and the ECG segment, respectively, are selected as our proposed intelligent SAA for the 8 s-segment length. The relatively high performance implies that the fully augmented ECG with its SH and NSH signals are reliable for the FE. Moreover, the FS algorithm is effective due to the small number of features in the FFC compared with the total ICFs.

**Table 6:** Five-folds CV of the ML classifiers using individual CFCs and CAF on the evaluation data

CFC	ML	Ac (%)	Se (%)	Sp (%)	BER (%)
CFC1	SVM	99.51±0.37	98.07±1.09	99.77±0.24	1.08±0.56
**CFC2**	**SVM**	**99**.**56**±**0.31**	**98**.**22**±**0.86**	**99**.**84**±**0.19**	**0**.**96**±**0.43**
CFC3	SVM	99.52±0.30	98.40±1.01	99.71±0.24	0.94±0.52
CFC4	SVM	99.39±0.42	98.15±0.87	99.69±0.32	1.08±0.47
CFC5	SVM	99.53±0.30	98.08±1.18	99.82±0.12	1.05±0.59
CFC6	KNN	99.42±0.38	98.40±0.88	99.68±0.27	0.96±0.46
CAF	SVM	99.41±0.33	98.35±0.98	99.74±0.21	0.95±0.48

The method is also implemented for 5 s-segment data. The FFC includes 4, 10, and 14 features extracted from the SH, NSH signals, and ECG segment, respectively. The highest validated performance is produced by the SVM classifier with 99.49%, 97.97%, 99.76%, and 1.13% for Ac, Se, Sp, and BER on the evaluation data.

Regarding the complexity, a proper interval consumed by the proposed ML-based SAA needs to be smaller than a segment length to grantee no delay for diagnosis between two consecutive segments. Hence, an average time consumed by the proposed SAA is computed for 50 consecutive 8 s-segments as 7.9 s including the time consumed for segment pre-processing, mode reconstruction, feature extraction, and classification. Moreover, an average time duration is 3.9 s, which is consumed by the SVM classifier using a combination of 28 features for 50 consecutive 5 s-segments. Table 7 compares the proposed SAA with representative candidates of the SAA using ML and DL techniques in terms of the SH/NSH rhythm classification performance.

**Table 7:** Comparison of our proposed SAA to representative candidates of individual categories

Refs.	Method							Performance					
	Signal preprocessing	Signal generation	Number of signals	FE	FS	Classification method	Validation method	Number of selected features	Classifier	Testing data			Average time consumed for 50 consecutive segments
										Ac (%)	Se (%)	Sp (%)	
[10]	Mean subtraction, Moving average, High-pass, Low-pass Butterworth filters	NA	1	TCSC	NA	Threshold based SAA	NA	1		98.1	80.9	98.5	NA
[44]	Moving average, High-pass, Low-pass Butterworth filters	Augmented ECG with MVMD	2	54 features with conventional methods	GA and SFFS	ML based SAA	Five-fold CV	20	SVM	99.0	97.4	99.2	4.7 s
									KNN	98.9	97.3	99.1	
									Boosting	98.4	97.4	98.5	
[8]	Moving average, High-pass, Low-pass Butterworth filters	Fully augmented ECG with MVMD	3	100 deep features with CNN	NA	DL based SAA	Five-fold CV	100	SVM	98.8	94.9	99.5	
									KNN	99.1	97.2	99.2	
									Boosting	99.3	97.1	99.4	7.0 s
Proposed algorithm	Moving average, High-pass, Low-pass Butterworth filters	Fully augmented ECG with MVMD	3	93 features with conventional methods	SFFS	ML based SAA	Five-fold CV	36	SVM	99.6	98.2	99.8	7.9 s
									KNN	99.3	97.7	99.9	
									Boosting	98.2	95.4	98.9	

## Research opportunities

Since the ML- and DL-based SAAs outperform the threshold-based counterparts, further researches are likely inclined to ML and DL techniques for applicable designs of the SAA in practical AEDs. Obviously, the SAA consists of various technical procedures including biomedical signal processing, feature engineering, and classification. Clearly, upgrading every single procedure and/or all such the procedures certainly result in the improvement of SAA diagnosis performance. Hence, the technical challenges exist definitely in all the steps of the method development for SAA designs. Indeed, the quality of processed ECG segments is limited by the use of low- and high-pass filters for the interference removal. Moreover, the improvement of conventional algorithms for the extraction of high quality features or new feature creation is the technical challenge for the construction of reliable SAAs. The optimal structures and parameters of the ML and DL methods also need to be addressed for better classification performance. However, this is a time-consuming process and may require a huge amount of hardware for simulation. To the best of our knowledge, the implementation and advancement of all technical procedures, as described in Fig. 2 for example, bring definitely on better research opportunities for the achievement of the optimal intelligent SAAs.

### Signal processing

The majority of publications covered in this paper use different signal processing techniques to improve the quality of signals and the extracted features such as low and high filtering. However, their limitations as insignificant improvement in the final classification performance of the SAAs have been observed. Therefore, the authors of [56] suggest a highly effective MVMD technique to remove the correlated properties of the input signal, which lead to the identifications, the suppression of ECG artifacts, and the enhancement of SAA detection performance. Indeed, consideration of the MVMD and ensembled EMD for the above works certainly opens up plenty of room for future research in investigating various signal processing techniques for the SAA designs.

### Feature engineering

The feature engineering contains the FE, FS, and activity to create newly informative features in an attempt to improve the final detection performance of the intelligent SAA design. Firstly, the development of intelligent SAA concentrates mainly on the investigation of the ICFs extracted by different FE methods and the FS algorithms to select the most relevant and informative FFC. Unfortunately, most of the well-known and exhaustive sets of ICFs have been explored. Therefore, a useful expansion of the ICFs extracted from fully augmented ECG with its SH and NSH signals has been investigated with a larger number of ML classifiers in a subsection of intelligent ML-based SAA. However, a common wide band of the AED employed for the construction of SH and NSH signals from the ECG segments results in a question that whether SH and NSH signals can be constructed on differently narrow frequency bands to select only SH and NSH components. That would be a good selection for future research due to the elimination of unuseful harmonics or frequencies which contributes probably to a decrease in the diagnosis performance of the SAA design.

Secondly, the chance to select a better FFC is improved by the use of various data transformation methods, which map the original ICFs into another space of ICFs. Then, the FS algorithms are applied for the selection of the most relevant and uncorrelated FFC in this space. Among transformation techniques, the principal component analysis is emerged as a promising method due to its ability to present uncorrelated and correlated characteristics of the original ICFs. Unfortunately, the data transformation has not been investigated adequately in existing publications.

The third important content of feature engineering is the creation of new features in the ICFs. Indeed, most previous studies related to the intelligent SAA design use the threshold-based methods as the ICFs. No newly relevant feature has been proposed in existing works. Hence, the computation of newly informative features should be considered seriously to enhance the feature quality and final detection performance of the SAA design.

### Update of the ML-based SAA

Currently, the ML classifiers require a large number of samples for training to improve the classification performance. However, this may cause an increment of complexity while reducing the learning effectiveness due to irrelevant samples, and a large number of support vectors. To overcome this problem, the authors of [57] suggest an efficient incremental learning method for the SVM including single-sample learning. In this work, the sample, which is added into training data, is checked whether it may have an impact on the SVM classifier by using the Karush-KuhnTucker conditions. If it does not satisfy these conditions, then the SVM classifier needs to be updated with new training data. Moreover, to optimize the number of useful support vectors, an adaptive pruning algorithm is proposed for the SVM in [58]. In this algorithm, the incremental and decremental learning procedures are implemented alternately to maintain a small scale of support vector set, which represents most of the information in the training set.

The increment learning procedure for the SVM can be applied for the design of intelligent SAA. Here, the single-segment learning procedure is implemented with the individual segments collected from the patient. This segment is checked its eligibility for adding into training data. Moreover, the advantage of using directly the collected segments is that patients have different characteristics in terms of SH/NSH rhythm, which can be exposed on the ECG signal. The classification performance of intelligent SAA for the AED can be improved if it can be trained on real-time data to fit in patient characteristics. The adaptive SVM, which is proposed in [55], is also useful to obtain a set of informative support vectors, which maintain an optimum number of samples while covering the most common characteristics of SH/NSH rhythm for SCA detection.

### Deep learning

The strong capabilities of the CNNs for feature learning without the requirements of conventional FE as well as feature ranking or feature scores for the FS make them ideal for being used as the feature extractor or full classification algorithms. However, the detection performance of the full CNN does not meet the AHA recommendations as shown in [7] while it is relatively high for the CNN used as the feature extractor in combination with ML classifiers according to [8, 46, 47]. It is supposed that the CNN is fitted in the use of feature extractor rather than that of a full detection method. On the other hand, the best CNN structure is selected by the exhaustive search algorithm, which results in the high complexity and time-consuming process, as shown in [8]. Clearly, other DL classifiers such as recurrent neural networks and recursive neural networks should be investigated for the SAA design in terms of SH/NSH rhythm classification improvement. Furthermore, the structure and parameters of the proposed DL algorithm should be selected carefully by using the different optimization methods on separate amounts of data to avoid the over-fitting problem and achieve the efficacy of the learning process.

## Conclusion

The threshold-based methods have been adopted for the FE and proven their effectiveness and simplicity for the design of SAAs. Basically, the requirement of expert human knowledge was demanded certainly for the extraction of the dominant, relevant features and the construction of efficient thresholds to classify SH/NSH rhythms. However, most of these methods reported a relatively low Se, which did not meet the AHA recommendations for AED performance. To cope with this obstacle, intelligent ML-based SAAs, which are designed with ML classifiers using a set of ICFs in which each ICF was an individual feature used for the threshold-based SAA designs, have been taken into account. Moreover, the DL techniques including CNN have been used to learn various characteristics of multiple input channels for the extraction of the deep ICFs, which are fed into ML classifiers. The classification performance of intelligent SAAs is better than that of threshold-based ones and complies with the AHA recommendations. Especially, the SAA using SVM classifiers and a set of features extracted from fully augmented ECG segment with its SH and NSH signals can obtain a significantly high performance, i.e. with the Se of 98.2% and the Sp of 99.8%. This developed SAA design proves that the fully augmented ECG signal is more suitable and effective for the FE of intelligent SAAs using ML and/or DL. It is obvious that the common target of the SAA development is to improve the classification performance. Yet, advanced signal processing techniques, feature engineering, and intelligent SAA design using ML and DL need to be further developed in terms of reliable designs for SH/NSH rhythm classification.

## Data Availability

Not applicable.

## Abbreviations

AED: Automated external defibrillator
AHA: American Heart Association
Ac: Accuracy
BER: Balance error rate
CAF: Combination of all ICFs
CFC: Candidate feature combination
CNN: Convolutional neural network
CNNE: Convolutional neural network extractor
CPR: Cardiopulmonary resuscitation
CUDB: Creighton University Ventricular Tachycardia database
CV: Cross validation
CWT: Continuous wavelet transform
DL: Deep learning
DNN: Deep neural network
DWT: Discrete wavelet transform
ECG: Electrocardiogram
EMD: Empirical mode decomposition
FE: Feature extraction
FFC: Final feature combination
FFEWT: Fixed frequency range empirical wavelet transform
FS: Feature selection
FV: Feature validation
GA: Genetic algorithm
GRAE: Gain ratio attribute evaluation
ICF: Input candidate feature
KNN: K-nearest neighbor
MITDB: MIT-BIH Arrhythmia database
ML: Machine learning
MVMD: Modified variational mode decomposition
NEWFM: Neural network with weighted fuzzy membership function
NSH: Non-shockable
NSRDB: Normal sinus rate data base
OHCA: Out-of-hospital-cardiac-arrest
SAA: Shock advice algorithm
SBFS: Sequential backward feature selection
SCA: Sudden cardiac arrest
Se: Sensitivity
SFFS: Sequential forward feature selection
SH: Shockable
Sp: Specificity
SVM: Support vector machine
RF: Random forest
VFDB: MIT-BIH Malignant Ventricular Arrhythmia database
VF: Ventricular fibrillation
VT: Ventricular tachycardia
VMD: Variational mode decomposition
